# Quantitative and Spatial Analysis of CD8+/PD-1 Tumor-Infiltrating Lymphocytes as a Predictive Biomarker for Clinical Response of Melanoma In-Transit Metastases to Topical Immunotherapy

**DOI:** 10.1245/s10434-020-08713-1

**Published:** 2020-06-15

**Authors:** Sophia Haywood, Jennifer Garioch, Arjun Ramaiya, Marc Moncrieff

**Affiliations:** 1 Norwich Medical School, University of East Anglia, Norwich, UK; 2 Department of Dermatology, Norfolk and Norwich University Hospital, Norwich, UK; 3 Cotman Centre of Cellular Pathology, Norwich Research Park, Norfolk and Norwich University Hospital, Norwich, UK; 4 Department of Plastic and Reconstructive Surgery, Norfolk and Norwich University Hospital, Norwich, UK

## Abstract

**Background:**

Melanoma in-transit metastases (ITMs) are a challenge to treat and associated with systemic disease and poor prognosis. Topical diphencyprone (DPCP), a potent contact sensitizer, is an established treatment for melanoma ITMs. This exploratory study investigated the utility of *BRAF* mutation status, CD8, PD-1, PD-L1, and TILs distribution as biomarkers for response of ITMs to topical immunotherapy (DPCP).

**Methods:**

The ITM deposits of 40 patients treated with DPCP were subjected to biomarker analysis for *BRAF* status, CD8 and PD-1 expression on tumor-infiltrating lymphocytes (TILs), and tumor PD-L1 expression. Response to DPCP and overall survival (OS) were compared by biomarker status.

**Results:**

After 12 weeks, 10 patients (25%) had a complete response, 12 patients (30%) had a partial response, and 18 patients (45%) had no response. No significant association was found between any individual biomarker and response to DPCP or OS. The *BRAF* mutation rate was 25% (10/40). All the patients with a complete response had *BRAF* wild-type tumor. Peritumoral CD8+ T-cells were associated with complete response (*P* = 0.041). Both CD8+ and PD-1 expressions were highly correlated (*P* < 0.0001), and the highest levels of PD-1 expression were detected at the peritumoral interface (*P* = 0.0004). Only two cases were PD-L1-positive, and both had a complete response to DPCP (*P* = 0.043).

**Conclusion:**

Patients who have *BRAF* wild-type tumor are more likely to experience a complete response to DPCP. Peritumoral TILs and PD-1 expressions may predict a better response to DPCP. Expression of PD-L1 may be associated with a complete response to DPCP. A larger prospective study is required.

In-transit metastases (ITMs) occur in up to 12% of patients with a diagnosis of cutaneous melanoma.^1^ The ITMs are locally recurrent deposits of melanoma located predominantly in the dermal and subdermal lymphatics between the primary and draining lymph nodes. In-transit metastases are challenging to treat, presenting heterogeneously from a single large lesion to multiple superficial papules (0.2–2 cm), erythematous or pigmented. The lesions continue to grow in size and number, eventually causing significant morbidity to patients.

Normally, ITMs occur in the first 36 months after diagnosis of the original primary tumor, and patients usually have no other distant metastases at the time of presentation.^1^,^2^ An ITM occurring in isolation represents advanced stage 3 disease according to the latest American Joint Committee on Cancer (AJCC) staging system for melanoma, with an overall 5-year survival rate of 32% to 83% and a 10-year survival rate of 24% to 77%.^3^ Many patients with ITMs are otherwise asymptomatic and survive for a significant period after presentation, which means that the lesions require effective palliation, treatment, or both.

Many treatment options are available for ITMs, ranging from simple ablative procedures (e.g., surgical excision or CO_2_ laser ablation) to complex locoregional therapies (e.g., limb infusion/perfusion or systemic therapy).^2^,^4^ Topical immunotherapy is a convenient, first-line therapy for patients with low-burden disease,^5^,^6^ and the agent of choice in our center is diphencyprone (DPCP).^7^

The topical immunotherapy agent, DPCP, was first reported as a treatment for melanoma metastases in 1989.^8^ As a hapten, DPCP elicits a CD8+ T-cell-mediated, delayed hypersensitivity response, which in turn stimulates the release of cytokines, specifically IL-24 and IL-9, which are known to act in melanoma as tumor-suppressor cytokines promoting lymphocyte-mediated tumor destruction.^9^

A potent contact sensitizer, DPCP causes delayed hypersensitivity reactions in 98% of people.^10^ Once sensitized, patients apply DPCP cream to lesions once weekly, inducing a local inflammatory reaction.^11^ Patients tolerate DPCP well with no systemic side effects. Our long-term outcomes with this therapy have been previously reported.^7^

The most prevalent gene identified in melanoma is the mutation of the proto-oncogene *BRAF*, present in 41% to 56% of all melanomas.^12^ This mutated protein is implicated in different means of melanoma progression, including activation of the MEK/ERK pathway, evading the immune response, senescence, and apoptosis as well as angiogenesis, tissue invasion, and metastasis.^13^,^14^ In 2010, *BRAF* mutation was first described as a therapeutic target and currently is the standard of care for suitable patients.^15^,^16^

Importantly, CD8+ cytotoxic T lymphocytes recognize tumor-associated antigens presented by tumor cells on their surface with major histocompatibility complex class 1 molecules (MHC I). Cytotoxic T cells demonstrate clinically significant anti-tumor activity in melanoma.^17^ Infiltration of CD8+, generally associated with improved survival, is a good prognostic marker.^18^ The programmed cell death 1 (PD-1) immune inhibitory receptor is present on T- and B-lymphocytes, natural killer cells, and myeloid cells, and PD-L1 is its corresponding ligand expressed on tumor cells, T and B cells, macrophages, and dendritic cells.^19^ The PD-1/PD-L1 interaction is a recognized mechanism whereby malignant cells evade the immune system to allow tumor growth and progression. High levels of PD-L1 expression in tumors are associated with a poorer prognosis.^20^ The blockade of the PD-1 pathway is performed in melanoma treatment with the use of systemic anti-PD1 therapy.^21^,^22^

The current study aimed to evaluate any link between the expression of *BRAF* mutation, CD8, PD-1, and PD-L1 in ITMs and clinical response to DPCP.

## Methods

Patients who had biopsy-proven ITMs treated with DPCP at our quaternary referral cancer center between 2008 and 2016 were identified from our institutional melanoma database. Patients with AJCC stage 3 melanoma at the time of presentation who had unresectable low-volume disease (superficial small metastatic deposits in the dermis and subcutis) were triaged to DPCP as first-line therapy by the assessing clinicians and the Specialist Multidisciplinary Team (MDT).

The treatment protocol for ITMs with DPCP has been previously described.^5^,^7^ In brief, the patients were initially sensitized to DPCP in the clinic, and once contact sensitivity was confirmed, the patients were started on a once-weekly regimen of 0.05% topical DPCP applied to the area under the direction of the treating clinician in the patient’s home. The patients were monitored every 2 weeks to gauge their clinical reaction to the DPCP, and the concentration of the agent was modified accordingly. The maintenance concentration of DPCP used varied from 0.000001 to 0.05%.

Clinical response to treatment was assessed and recorded by the senior clinician (J.J.G.) in a combined MDT clinic, in which a contemporaneous second opinion from other clinicians was available if the lack of clinical response was concerning or other simultaneous treatment methods were needed (e.g., surgical excision, CO_2_ laser ablation, or systemic therapy) in case of a partial response. Clinical responses were recorded 3 and 6 months after the initiation of treatment with DPCP and graded CR (complete response with no viable tumor observed), PR (partial response with some, but not all, of the tumor resolved), or NR (none, with none of the presenting ITMs showing a response to the DPCP or with the development of more ITMs and no evidence of a response). The total response rate was calculated as CR + PR/all patients × 100%. Treatment was continued for at least 12 months if the patient responded or discontinued if the disease showed obvious uncontrolled progression.

### Immunohistochemistry and Molecular Diagnostics

Archived formalin-fixed, paraffin-embedded surgical histology specimens of in-transit metastases were retrieved. Histology was reviewed on hematoxylin and eosin (H&E) slides, and a single representative block was chosen for immunohistochemistry (IHC). Before the start of DPCP treatment, 34 specimens (85%) were taken, and 6 further samples (15%) were taken afterward.

All IHC was performed on an Leica bond immunostainer (Leica Microsystems (UK) Ltd, Milton Keynes, UK) according to the manufacturer’s instructions. Testing of *BRAF* mutation was performed according to our clinical standard of care, which has been published elsewhere.^23^ For *BRAF* V600E IHC, Pleasanton, CA Spring Bioscience mouse anti-human *BRAF* V600E monoclonal antibody (Clone VE1) was used at a dilution of 1:200 run on a routine long program.

The *BRAF* V600E IHC was reported as positive or negative. Any negative specimens or specimens displaying variable expression underwent molecular diagnostic testing (Biocartis Idylla system; PCR, Mechelen, Belgium) to identify other *BRAF* V600 mutations.

For CD8 IHC, Leica 4B11 clone CD8-4B11-L-CE was used at 1:40 dilution on a routine heat retrieval program. For PD-1 IHC, Cell Marque PD-1 EP239 (315R-14), Rocklin, CA was used at 1:250 dilution on a routine heat retrieval program. Positive control for CD8 and PD-1 included tonsillar tissue. For PD-L1 IHC, Cell Marque PD-L1 28-8 (438R-14-ASR) was used at 1:75 dilution on a long heat-retrieval program. We used PD-L1-positive cell lines, and positive control included endometrial and tonsillar tissue. Every batch of IHC was run with a satisfactory control line.

Expression of CD8 and PD-1 on positive TILs within tumor nests was reported per square millimeter in the area of the most dense lymphocyte infiltration at high-power (×40) fields. The counts per square millimeter were grouped into the following levels of expression: 0 (0 cells/mm^2^), 1 (1–10 cells/mm^2^), 2 (11–100 cells/mm^2^), and 3 (≥ 101 cells/mm^2^). Expression of PD-L1 was seen on tumor cells and immune cells. To provide percentage of expression, PD-L1-positive tumor cells were counted, and 5% or higher was considered as indicating PD-L1 positivity.

Detection and observer bias was avoided in this study due to blinding of researchers analyzing the specimen slides to any clinical data.

### Spatial Distribution of CD8+ TILs

**Fig. 1:**
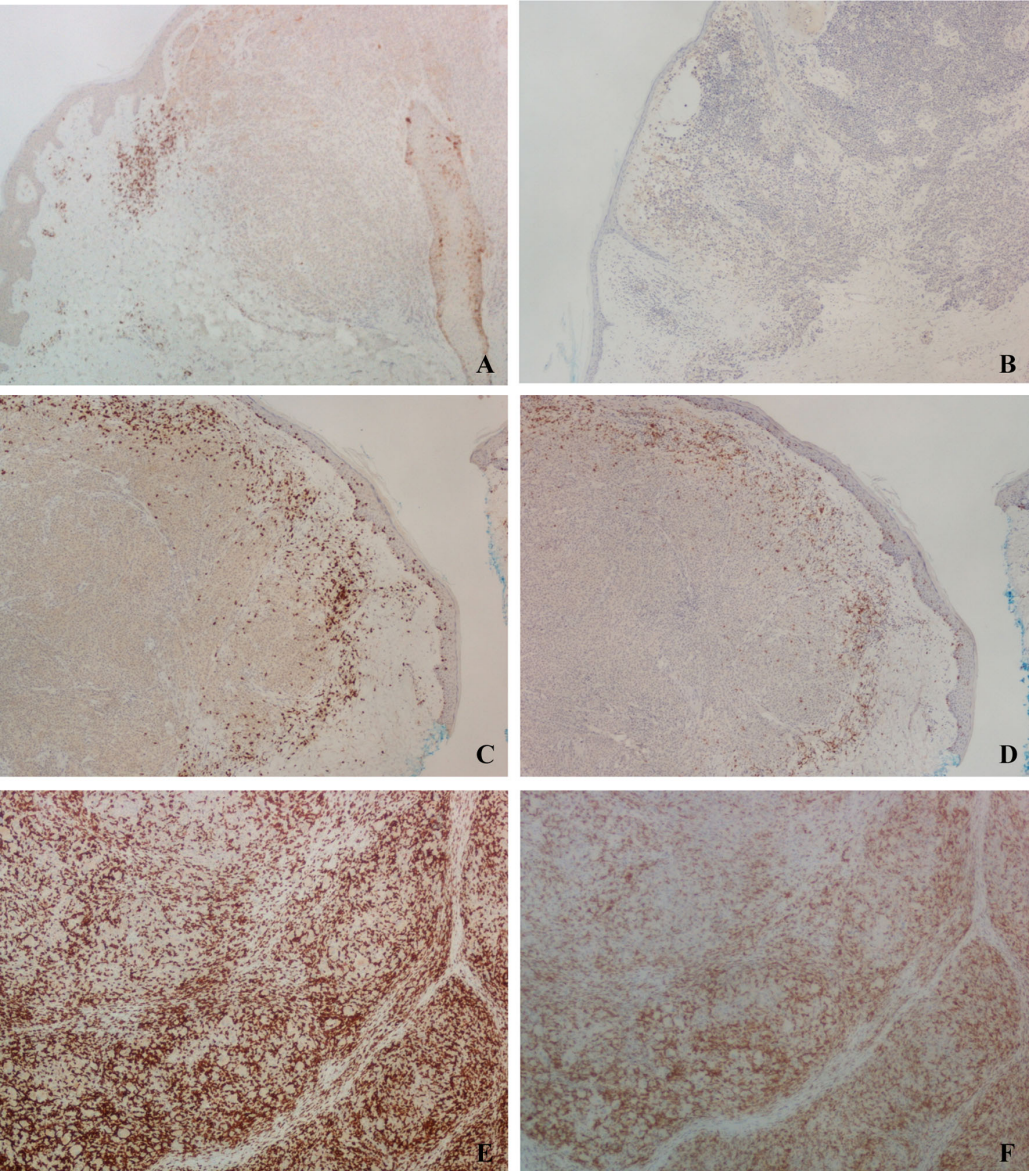
A CD8+ peripheral/absent TILs (× 1); B peripheral/absent PD-1 TILs (× 1); C CD8+ peritumoral TILs (× 1); D PD-1+ peritumoral TILs (× 1); E CD8+ intratumoral TILs (× 1); F PD-1+ intratumoral TILs (× 1)

The location of the TILs in relation to the tumor deposit was assessed using both the H&E stains and CD8 immunostains and classified into three distinct patterns based on the distribution of the CD8+ lymphocytes as follows: peripheral/absent (lymphocytes predominantly located in the perivascular region or absent altogether), peritumoral (lymphocytes predominantly located at the tumor edge/margin or tumor-stroma interface), or intratumoral (lymphocytes predominantly located within the tumor, with few or no lymphocytes in the peritumoral location). Figure 1 shows representative examples of each pattern.

### Statistical Analysis

Biomarker status was tabulated by response group. Chi square tests and Fisher’s Exact Test were used to establish association between biomarker status and response to DPCP. The Kaplan-Meier log-rank method was used for survival estimates and graphic representation. Analysis was performed with GraphPad Prism v. 8.0 (GraphPad.com, San Diego, CA, USA).

## Results

**Fig. 2:**
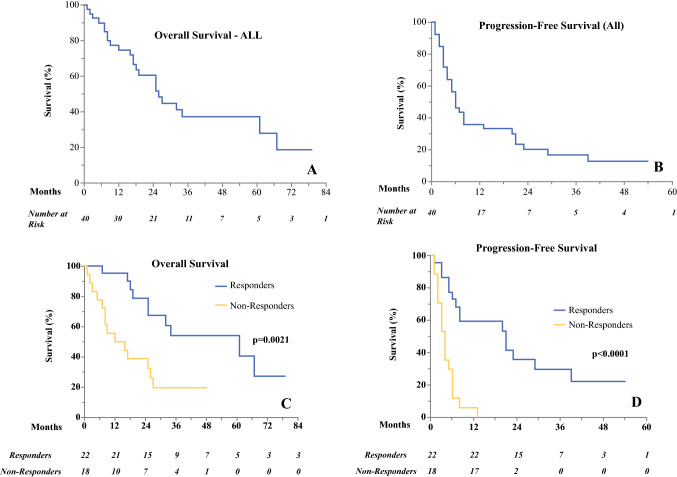
**a** Overall survival. **b** Progression-free survival. **c** Comparison of OS between responders and non-responders. **d** Comparison of PFS between responders and non-responders

Ethics approval was granted for this study (IRAS ID: 227816; Cambridge Regional Ethics 17/EE/0451). The cohort of 40 patients treated between 2008 and 2016 with samples eligible for biomarker analysis included 22 women (55%) and 18 men (45%). The cohort had a median age of 76 years (range, 47–95 years). After 12 weeks of treatment with DPCP, 10 patients (25%) had CR, 12 had PR (30%), and 18 (45%) had NR, for an overall response (CR+PR) of 55%. The median OS was 26 months (range, 2–79 months) (Fig. 1a). During the study period, 24 patients died. The median progression-free survival (PFS) was 6 months (Fig. 2b). A clinical response to DPCP was significantly associated with increased OS (*P* = 0.0021) and PFS (*P* < 0.0001) (Fig. 2c and d) (Table 1).

**Table 1:** Response to DPCP correlated with OS and PFS

Response	*n* (%)	Median OS Months (IQR)	Deaths (*n*)	HR (95% CI)	*P* Value^a^
NR					
CR or PR	18 (45)	14.0 (7.0– 27.0)	14	0.3153	0.0021
	22 (55)	61.0 (25.0–NtR)	10	(0.132–0.751)	

Response	*n* (%)	Median PFS Months (IQR)	Recurrences (*n*)	HR	*P* Value
NR response					
CR or PR	18 (45%)	4.0 (2.0–5.5)	18	0.2703	<0.0001
	22 (55%)	21.0 (6.0–34.0)	15	(0.119–0.612)	

*DPCP* diphencyprone, *OS* overall survival, *PFS* progression-free survival, *IQR* interquartile range, *HR* hazard ratio, *NR* no response, *PR* partial response, *CR* complete response, *NtR* not reached

^a^Log-rank test

### Biomarker Distribution and Analysis

All identified *BRAF* mutations were of the V600E subtype. The 40 specimens comprised 10 (25%) *BRAF* V600E mutation-positive and 30 (75%) *BRAF* mutation–wild-type specimens. Table 2 shows the distribution of the lymphocyte biomarkers according to *BRAF* status. No significant association was observed between *BRAF* mutation status and CD8+, PD-1, or PD-L1 counts. The distribution of the TILs was not associated with *BRAF* status.

**Table 2:** Lymphocyte biomarkers and distribution by BRAF mutation status

Variable	Category	BRAF wild type (*n* = 30)	BRAF V600E (*n* = 10)	Total *n* (%)	*P* Value^a^
CD8+	< 1	3	3	6 (15)	0.3186
(cells/mm^2^)	1–10	12	2	14 (35)	
	11–100	9	4	13 (32.5)	
	> 100	6	1	7 (17.5)	
PD-1	< 1	12	3	15 (37.5)	0.8281
(cells/mm^2^)	1–10	10	5	15 (37.5)	
	11–100	4	1	5 (12.5)	
	> 100	4	1	5 (12.5)	
Predominant TILs	Absent/peripheral	9	3	12 (30)	0.6778
Distribution	Peritumoral	10	2	12 (30)	
	Intratumoral	11	5	16 (40)	
Tumor PD-L1	≤ 5%	28	10	38 (95)	0.4020
	> 5%	2	0	2 (5)	

*PD-1* programmed cell death 1, *TILs* tumor-infiltrating lymphocytes, *PD-L1* programmed death-ligand 1

^a^Fisher’s Exact Test

### Lymphocyte Biomarkers and TILs Distribution

Table 3 shows the biomarker TILs distribution analyses. A strong association with PD-1 and CD8 counts was observed (*P* < 0.0001, Fisher’s Exact Test). A Chi square test for trend indicated a significant association between the two-variables (*P* < 0.0001). The predominant TILs patterns were evenly distributed among the three subgroups. The PD-1 cell counts were significantly associated with TILs distribution (*P* = 0.0004). A significant trend toward the peritumoral TILs pattern was observed, demonstrating higher PD-1 cell counts (*P* = 0.0012). Tumors with predominantly peritumoral TILs demonstrated high PD-1 cell counts (> 10 cells/mm^2^) in more than half (58.3%) of the specimens analyzed, whereas the PD-1 cell counts in the absent/peripheral and intratumoral TILs groups were low (≤ 10 cells/mm^2^) for the majority of specimens (16.7% and 6.2%, respectively; *P* = 0.006).

**Table 3:** CD8+ lymphocyte count, TILs location, and PD-1 biomarker status

VARIABLE	Category	PD-1 (cells/mm^2^)					
		< 1	1–10	11–100	> 100	Total *n* (%)	*P* Value
CD8+ count	< 1	4	1	1	0	6 (15)	
(cells/mm^2^)	1–10	9	5	0	0	14 (35)	< 0.0001^a^
	11–100	2	8	3	0	13 (32.5)	< 0.0001^b^
	> 100	0	1	1	5	7 (17.5)	
TILs location	Absent/peripheral	8	2	2	0	12 (30)	
	Peritumoral	0	5	2	5	12 (30)	0.0004^a^
	Intratumoral	7	8	1	0	16 (40)	0.0012^b^
		Low PD-1 (≤ 10 cells/mm^2^) *n* (%)		High PD-1 (> 10 cells/mm^2^) *n* (%)			
TILs location	Absent/peripheral	10 (83.3)		2 (16.7)		12 (30%)	
	Peritumoral	5 (41.7)		7 (58.3)		12 (30%)	0.0060^a^
	Intratumoral	15 (93.8)		1 (6.2)		16 (40%)	0.0051^b^

*TILs* tumor-infiltrating lymphocytes, *PD-1* programmed cell death 1

^a^Fisher’s Exact Test

^b^Chi square test for trend

### Response to DPCP and Biomarker Status

All the patients with a CR had a *BRAF* wild-type tumor (Table 4). The presence of a *BRAF* V600E mutation was associated with a poor clinical response (*P* = 0.025). The responding groups (CR+PR) showed a trend for higher overall CD8+ counts, but this was not statistically significant (*P* = 0.165). Increasing PD-1 cell counts were associated with a better response to DPCP (*P* = 0.042). Although only two tumors demonstrated measurable PD-L1, the presence of this receptor was significantly associated with a CR (*P* = 0.043). Table 5 shows that although no significant association was demonstrated with CD8+ TILs distribution and response to DPCP (*P* = 0.1281), subanalysis showed that peritumoral CD8+ TILs were associated with a favorable response to DPCP (*P* = 0.0421). Similarly, 60% of the CRs were associated with tumors that demonstrated this distribution, which was significant (*P* = 0.0410). Other patterns of CD8+ TILs distribution were not predictive of DPCP response. A low PD-1 cell count (≤ 10 cells/mm^2^) was associated with significantly low complete response rates compared to diphencyprone (low rates of CR to DPCP) compared with a high PD-1 cell count (13.3% vs 60%; *P* = 0.0061).

None of the biomarkers investigated in this study were associated with overall survival (OS) of the patients in this cohort (Table 4).

**Table 4:** Clinical response to DPCP correlated with OS by biomarker status

Variable	Category	CR *n*	PR *n*	NR *n*	Total *n* (%)	*P* Value^a^	Median OS Months (IQR)	Deaths (*n*)	*P* Value^b^
BRAF	Wild type	10	6	14	30 (75)	0.025	25.0 (9.0–39.0)	17	0.412
	V600E	0	6	4	10 (25)		17.5 (14.0–26.0)	7	
CD8+	< 1	1	3	2	6 (15)	0.165	18.5 (7.0–49.0)	3	0.752
(cells/mm^2^)	1–10	3	2	9	14 (35)		25.0 (9.0–30.0)	11	
	11–100	2	6	5	13 (32.5)		25.0 (14.0–34.0)	8	
	> 100	4	1	2	7 (17.5)		17.0 (10.0–26.0)	2	
PD-1	< 1	1	5	9	15 (37.5)	0.042	25.0 (8.0–44.0)	10	0.706
(cells/mm^2^)	1–10	3	4	8	15 (37.5)		20.0 (14.0–32.0)	10	
	11–100	2	2	1	5 (12.5)		34.0 (18.0–49.0)	3	
	> 100	4	1	0	5 (12.5)		17.0 (15.0–17.0)	1	
Tumor PD-L1	≤ 5%	8	12	18	38 (95)	0.043	19.5 (10.0–32.0)	22	0.919
	> 5%	2	0	0	2 (5)		47.5 (34.0–61.0)	2	

*DPCP* diphencyprone, *OS* overall survival, *CR* complete response, *PR* partial response, *NR* no response, *IQR* interquartile range, *PD-1* programmed cell death 1, *PD-L1* programmed death-ligand 1

^a^Chi square test for trend

^b^Log-rank test

**Table 5:** Predominant CD8+ TILs location subanalysis and response to DPCP

Variable	Category	CR (*n*)	PR (*n*)	NR (*n*)	*P* Value
*Lymphocyte response*					
TILs location	Absent/peripheral	3	3	6	0.1281^a^
	Peritumoral	6	3	3	
	Intratumoral	1	6	9	
TILs location	Peritumoral	6	3	3	0.0421^a^
(subanalysis)	Peripheral or intratumoral	4	9	1	0.0172^b^

Variable	Category	CR	PR or NR	*P* Value
TILs location	Peritumoral	6	6	0.0410^a^
(subanalysis)	Peripheral or intratumoral	4	24	

Variable	Category	CR	PR	NR	*P* Value
PD-1	Low (≤ 10)	4	9	17	0.0061^a^
(cells/ mm^2^)	High (> 10)	6	3	1	0.0062^b^
(All TILs)					
PD-1	Low (≤ 10)	1	1	3	0.0985^a^
(cells/mm^2^)	High (> 10)	5	2	0	0.0542^b^
(peritumoral TILs)					

*TILs* tumor-infiltrating lymphocytes, *DPCP* diphencyprone, *CR* complete response, *PR* partial response, *NR* no response, *PD-1* programmed cell death 1

^a^Chi square test

^b^Chi square test for trend

## Discussion

The response to DPCP and the survival rates are in keeping with the results published by the current group,^7^ two Australian cohorts,^5^,^6^ and two smaller Canadian and Brazilian cohorts.^24^,^25^ Collation of the published data for 192 patients to date shows that DPCP has an overall response (CR + PR) rate of 64.6%. The findings showed an overall CR of 30.7% (range, 13–46%), an overall PR of 33.9% (range, 25–38.9%), and an NR rate of 35.4% (range, 18–60%).

### BRAF V600E Mutation Status

Read et al.^6^ reported a *BRAF* V600 mutation rate of 26.9% in a similar cohort of patients with ITMs but did not comment in its relation to DPCP response. This is close to the current study’s positive mutation rate of 25%. These two independent cohorts of patients with ITMs demonstrate a lower rate of *BRAF* positivity than the expected rate of 50% for all melanomas. One interpretation maintains that *BRAF* V600 mutant tumors are less likely to develop ITMs, although further cohorts are required to verify this. Adler et al.^26^ reported that in *BRAF*-mutant patients, lymph node metastases are more likely to develop as first metastases than as in-transit metastases but do not report the rate of *BRAF* mutation in their group with ITMs. In this study, all 10 patients with a CR were *BRAF* wild type (*P* = 0.025), although *BRAF* status was not significantly associated with OS or PFS (*P* = 0.43). Notably, targeted systemic therapy was historically unavailable to most of the patients in our cohort. Therefore, it is not possible to draw any reasonable conclusions from our data alone. Other studies have reported that a *BRAF* V600 mutation is associated with higher metastatic burden, suggested that it is an independent negative prognostic factor for OS and distant metastasis-free survival.^27^^–^29

### CD8+ PD-1 TILs and the Tumor Microenvironment

A significant presence of T cells identified in the tumor microenvironment (TME) generally is associated with a good prognosis.^18^ In particular, CD8+ TILs are associated with improved RFS and OS for melanoma, which may be closely related to the directly killing effect of CD8+ TILs on tumor cells.^30^ The TME consists of cancer cells, inflammatory cells, stromal cells, and cytokines, and these components form a complicated immunosuppressive network in cancer, which limits T cell activation and induces T cell dysfunction. The PD-L1/PD-1-signaling pathway is a crucial regulatory pathway of T cell exhaustion in cancer, and PD-L1 is abundantly expressed in cancer cells and stromal cells. A marker of T cell exhaustion is the upregulation of the PD-1 receptor.^31^

Our results after simple profiling of the TME of ITMs, highlight the complexity of these interactions. Unlike previous TILs studies of primary melanoma, our data did not show a quantitative relationship between CD8+ T-cell count and response to therapy or OS. However, qualitative analysis of the TILs distribution showed that the predominant presence of CD8+ T cells at the stromal-tumor interface of the ITM is associated with significantly increased rates of response to the topical immunotherapy. Similarly, our data demonstrate that at that same location, a significantly greater proportion of PD-1 CD8+ cells exist, suggesting underlying mechanisms of localized immunosuppression at the stromal-tumor interface of the ITM that promote T cell exhaustion and prevent tumor destruction.^31^,^32^ Given that a delayed hypersensitivity reaction produced by the application of DPCP generates a dramatic influx of CD8+ cytotoxic T cells local to the ITMs,^9^,^33^ we hypothesize that the mechanism of the topical immunotherapy action is to overwhelm the TME with active CD8+ T-cells, thereby shifting the balance in favor of tumor destruction rather than PD-L1/PD-1 immunosuppression at the tumor-stromal interface.

Conversely, ITMs located in an immunologically “bland” TME associated with the BRAF V600E mutation and characterized by absent or predominantly intra-tumoral T cells and low PD-1 expression did not benefit from the localized inflammation induced by the hypersensitivity reaction and did not respond to DPCP. We noted with interest that in addition to the pooled international data, the proportionate distribution of CD8+ TILs closely mirrored the rate of the response to DPCP in our study, although this may have been coincidental and requires much investigation.

Notably, the rate of response to DPCP was heavily correlated with PFS (*P* = 0.0021) and OS (*P* < 0.0001) (Table 1), which raises the possibility that the same TME immune interactions are mirrored at metastatic sites. Correlation studies with systemic immunotherapy outcomes would be clinically useful because this would indicate that ITMs could potentially be studied as a translational model for systemic response to novel therapies.

### PD-L1 Expression

The result of two PD-L1-positive cases is surprising because PD-L1 expression is reported in up to 40% of melanoma cases, in both primary and metastatic lesions.^20^,^34^,^35^ The data from a recent meta-analysis of multiple solid tumors showed that PD-L1 overexpression is associated with worse disease-free and progression-free survival in melanoma (hazard ratio [HR],  3.39; 95% confidence interval [CI],  2.02–5.69; *P* < 0.0001).^36^

### Study limitations

This study had several limitations including its small sample size and possible selection bias in a non-randomized cohort. In particular, the multifocal and heterogeneous nature of the condition meant that the true burden of the disease could not be accurately measured or classified and that the single biopsy analyzed may not have been reflective for all the ITMs in the region. In addition, measurement of clinical response is necessarily subjective and has historically been an issue common for many studies investigating treatment for ITMs.^4^,^37^,^38^

Regarding the analyses, this was an exploratory study with limited resources. Therefore, our T cell subset was limited to CD8. The study would have been improved by additional T cell marker analyses, particularly analyses of CD3, CD4, and CD31, with cytokine assays to complement this work. Our sample was relatively small, and although we demonstrated significant findings in subanalyses, the group sizes were smaller, and the findings in this study should perhaps not be overstated. However, significant findings in small samples are challenging to achieve and, as such, the results appear to be compelling.

Finally, the results of patient outcomes were collated mostly in a period before the general availability of systemic immunotherapy. It would be interesting in future studies to correlate the response to local immunotherapy with the response to systemic immunotherapy.

## Conclusion

The study findings indicate that the tumor microenvironment plays an important role in determining the response of melanoma ITMs to topical DPCP. The results indicate that the absence of a *BRAF* V600 mutation and PD-L1 tumor expression are associated with a favorable response to DPCP, and qualitative assessment of CD8+ TILs may be useful for predicting the clinical outcome with DPCP. These results may be useful for clinicians triaging the sequence of treatments for low-burden disease. A larger prospective study is needed for further elucidation of the mechanisms whereby topical DPCP leads to resolution of melanoma ITMs in the skin.

## References

[1] Read RL, Haydu L, Saw RPM (2015). In-transit melanoma metastases: incidence, prognosis, and the role of lymphadenectomy. Ann Surg Oncol..

[2] Testori A, Ribero S, Bataille V (2017). Diagnosis and treatment of in-transit melanoma metastases. Eur J Surg Oncol..

[3] Gershenwald JE, Scolyer RA, Hess KR, et al. Melanoma staging: evidence-based changes in the American Joint Committee on Cancer eighth edition cancer staging manual. *CA Cancer J Clin*. 2017;67:472–92. 10.3322/caac.21409 PMC5978683 29028110

[4] Kroon HM, Moncrieff M, Kam PCA, Thompson JF (2008). Outcomes following isolated limb infusion for melanoma: a 14-year experience. Ann Surg Oncol..

[5] Damian DL, Saw RPM, Thompson JF (2014). Topical immunotherapy with diphencyprone for in-transit and cutaneously metastatic melanoma: DPCP for melanoma. J Surg Oncol..

[6] Read T, Webber S, Tan J (2017). Diphenylcyclopropenone for the treatment of cutaneous in-transit melanoma metastases: results of a prospective, non-randomized, single-centre study. J Eur Acad Dermatol Venereol..

[7] Moncrieff M, Fadhil M, Garrioch J (2016). Topical diphencyprone for the treatment of locoregional intralymphatic melanoma metastases (LIMMs) of the skin: the 5-Year Norwich experience. Br J Dermatol..

[8] Harland CC, Saihan EM (1989). Regression of cutaneous metastatic malignant melanoma with topical diphencyprone and oral cimetidine. Lancet..

[9] Gulati N, Suárez-Fariñas M, Fuentes-Duculan J (2014). Molecular characterization of human skin response to diphencyprone at peak and resolution phases: therapeutic insights. J Invest Dermatol..

[10] Van der Steen PH, Happle R (1993). Topical immunotherapy of alopecia areata. Dermatol Clin..

[11] Damian DL, Thompson JF (2007). Treatment of extensive cutaneous metastatic melanoma with topical diphencyprone. J Am Acad Dermatol..

[12] Lee J-H, Choi J-W, Kim Y-S (2011). Frequencies of BRAF and NRAS mutations are different in histological types and sites of origin of cutaneous melanoma: a meta-analysis. Br J Dermatol..

[13] Davies H, Bignell GR, Cox C (2002). Mutations of the BRAF gene in human cancer. Nature..

[14] Long GV, Menzies AM, Nagrial AM (2011). Prognostic and clinicopathologic associations of oncogenic BRAF in metastatic melanoma. J Clin Oncol..

[15] Flaherty KT, Puzanov I, Kim KB (2010). Inhibition of mutated, activated BRAF in metastatic melanoma. N Engl J Med..

[16] Robert C, Grob JJ, Stroyakovskiy D (2019). Five-year outcomes with dabrafenib plus trametinib in metastatic melanoma. N Engl J Med..

[17] Hadrup S, Donia M, Thor Straten P (2013). Effector CD4 and CD8 T cells and their role in the tumor microenvironment. Cancer Microenviron..

[18] Gooden MJM, de Bock GH, Leffers N, Daemen T, Nijman HW (2011). The prognostic influence of tumour-infiltrating lymphocytes in cancer: a systematic review with meta-analysis. Br J Cancer..

[19] Okazaki T, Honjo T (2007). PD-1 and PD-1 ligands: from discovery to clinical application. Int Immunol..

[20] Obeid JM, Erdag G, Smolkin ME (2016). PD-L1, PD-L2, and PD-1 expression in metastatic melanoma: correlation with tumor-infiltrating immune cells and clinical outcome. Oncoimmunology..

[21] Robert C, Ribas A, Schachter J (2019). Pembrolizumab versus ipilimumab in advanced melanoma (KEYNOTE-006): post hoc 5-year results from an open-label, multicentre, randomised, controlled, phase 3 study. Lancet Oncol..

[22] Ascierto PA, Long GV, Robert C (2019). Survival outcomes in patients with previously untreated BRAF wild-type advanced melanoma treated with nivolumab therapy: three-year follow-up of a randomized phase 3 trial. JAMA Oncol..

[23] Lo MCI, Paterson A, Maraka J (2016). A UK feasibility and validation study of the VE1 monoclonal antibody immunohistochemistry stain for BRAF-V600E mutations in metastatic melanoma. Br J Cancer..

[24] Rubinstein JC, Sznol M, Pavlick AC (2010). Incidence of the V600K mutation among melanoma patients with BRAF mutations, and potential therapeutic response to the specific BRAF inhibitor PLX4032. J Transl Med..

[25] Gibbons IL, Sonagli M, Bertolli E, Macedo MP, de Pinto CAL, Duprat Neto JP. Diphencyprone as a therapeutic option in cutaneous metastasis of melanoma. a single-institution experience. *An Bras Dermatol*. 2018;93:299–301. 10.1590/abd1806-4841.20187162 PMC5916415 29723355

[26] Adler NR, Wolfe R, Kelly JW (2017). Tumour mutation status and sites of metastasis in patients with cutaneous melanoma. Br J Cancer..

[27] Moreau S, Saiag P, Aegerter P (2012). Prognostic value of BRAF(V^600^) mutations in melanoma patients after resection of metastatic lymph nodes. Ann Surg Oncol..

[28] Picard M, Pham Dang N, D’Incan M (2014). Is BRAF a prognostic factor in stage III skin melanoma? A retrospective study of 72 patients after positive sentinel lymph node dissection. Br J Dermatol..

[29] Barbour AP, Tang YH, Armour N (2014). BRAF mutation status is an independent prognostic factor for resected stage IIIB and IIIC melanoma: implications for melanoma staging and adjuvant therapy. Eur J Cancer..

[30] Fu Q, Chen N, Ge C (2019). Prognostic value of tumor-infiltrating lymphocytes in melanoma: a systematic review and meta-analysis. Oncoimmunology..

[31] Jiang Y, Li Y, Zhu B (2015). T-cell exhaustion in the tumor microenvironment. Cell Death Dis..

[32] Ahn E, Araki K, Hashimoto M (2018). Role of PD-1 during effector CD8 T cell differentiation. Proc Natl Acad Sci U S A..

[33] Kalish RS, Askenase PW (1999). Molecular mechanisms of CD8+ T cell-mediated delayed hypersensitivity: implications for allergies, asthma, and autoimmunity. J Allergy Clin Immunol..

[34] Kaunitz GJ, Cottrell TR, Lilo M (2017). Melanoma subtypes demonstrate distinct PD-L1 expression profiles. Lab Invest..

[35] Taube JM, Anders RA, Young GD, et al. Co-localization of inflammatory response with B7-h1 expression in human melanocytic lesions supports an adaptive resistance mechanism of immune escape. *Sci Transl Med*. 2012;4:127ra37. 10.1126/scitranslmed.3003689 PMC3568523 22461641

[36] Wang Q, Liu F, Liu L (2017). Prognostic significance of PD-L1 in solid tumor: an updated meta-analysis. Medicine..

[37] Squires MH, Delman KA (2013). Current treatment of locoregional recurrence of melanoma. Curr Oncol Rep..

[38] Louie RJ, Perez MC, Jajja MR (2019). Real-world outcomes of talimogene laherparepvec therapy: a multi-institutional experience. J Am Coll Surg..

